# Functional heterogeneity of mast cells in cutaneous inflammation: implications for precision medicine

**DOI:** 10.3389/fimmu.2025.1680574

**Published:** 2025-10-14

**Authors:** Surui A, Dugarmaa Ulzii, Enkhtur Yadamsuren, Jihai Shi

**Affiliations:** ^1^ Department of Dermatology, The First Affiliated Hospital of Baotou Medical College, Inner Mongolia University of Science and Technology, Baotou, China; ^2^ Department of Dermatology, School of Medicine, Mongolian National University of Medical Sciences, Ulaanbaatar, Mongolia; ^3^ Baotou Medical College, Inner Mongolia University of Science and Technology, Baotou, China; ^4^ Department of Dermatology, Ordos Central Hospital, Ordos, China

**Keywords:** mast cells, inflammatory skin diseases, MRGPRX2, neuroimmune interaction, precision medicine

## Abstract

Inflammatory skin diseases, including atopic dermatitis, psoriasis, and chronic spontaneous urticaria, substantially impair patients’ quality of life. Despite therapeutic advances, current treatments often fail to achieve durable remission, underscoring the need for more precise interventions. Mast cells (MCs), traditionally recognized for their roles in IgE-mediated allergic responses, exhibit marked functional heterogeneity that shapes their pathogenic contributions to chronic skin inflammation. Recent single-cell and spatial transcriptomic analyses have identified discrete MC subsets with distinct inflammatory signatures and tissue-specific distributions, highlighting the complexity of their regulation within disease-specific microenvironments. A key mediator of non-IgE-dependent activation is Mas-related G protein–coupled receptor X2 (MRGPRX2), which engages diverse ligands and triggers receptor-biased signaling pathways, thereby promoting pathological neuroimmune interactions. Although MRGPRX2-targeted small molecules and antibodies have shown preclinical potential, major translational challenges remain, including the limitations of existing animal models and the lack of validated biomarkers. This review delineates MC heterogeneity, summarizes recent insights into MRGPRX2-mediated mechanisms, critically appraises current precision-targeted therapeutic strategies, and proposes solutions to overcome translational barriers. It is suggested that integrating advanced humanized models, longitudinal multi-omics profiling, and standardized functional assays may accelerate clinical translation and support the development of MC-targeted precision medicine.

## 1 Introduction

According to the Global Burden of Disease Study 2021, skin diseases rank as the seventh leading cause of disability worldwide (1). Among these, the global prevalence of atopic dermatitis (AD) has been estimated at 129 million and continues to rise (2). Inflammatory dermatoses such as AD, psoriasis (PsO), chronic urticaria (CU), and its subtype chronic spontaneous urticaria (CSU) are frequently characterized by refractory pruritus and pain. These symptoms substantially impair sleep quality, emotional well-being, and social functioning and are associated with a higher risk of psychiatric comorbidities, including anxiety and depression (3–5). Consequently, they represent key contributors to the reduced quality of life observed in affected individuals. Despite the clinical availability of second-generation antihistamines, glucocorticoids, and biologics targeting IL-4Rα (e.g., dupilumab) or IgE (e.g., omalizumab), significant therapeutic limitations persist. Standard-dose antihistamines demonstrate limited efficacy in CSU, with approximately 40% of patients failing to achieve symptom control even at fourfold increased doses (6). Although omalizumab is effective in a subset of patients, symptom recurrence is common after treatment discontinuation. The absence of validated biomarkers to predict relapse risk limits its utility as a stand-alone option for long-term disease management (6). These limitations underscore the need for a more comprehensive understanding of the underlying pathogenesis of inflammatory dermatoses. Recent investigations have therefore renewed focus on mast cells (MCs), which are increasingly recognized as central effector cells with substantial functional heterogeneity in the context of skin inflammation.

Recent advances in single-cell RNA sequencing (scRNA-seq) and spatial profiling technologies have revealed pronounced phenotypic and functional heterogeneity among MCs within the cutaneous microenvironment. This heterogeneity is reflected in the variable expression of inflammatory mediators (e.g., cytokines, chemokines, proteases), differential responsiveness to stimuli, distinct tissue localization, and migratory behavior (7). These insights have provided a conceptual foundation for exploring non-IgE-mediated activation pathways in skin inflammation. MC activation is now recognized to extend beyond the classical IgE–FcϵRI axis, involving diverse receptor interactions and complex intracellular signaling networks. Among these, Mas-related G protein–coupled receptor X2 (MRGPRX2) has been identified as a central mediator of non-IgE-dependent MC activation, due to its uniquely negatively charged ligand-binding pocket. MRGPRX2 is critically implicated in drug-induced pseudoallergic reactions, also referred to as atypical hypersensitivity, especially those triggered by neuromuscular blocking agents and fluoroquinolone antibiotics, as well as in neurogenic pruritus (8). Recent progress in high-resolution structural analyses (e.g., cryo-electron microscopy) and the development of selective small-molecule antagonists has established a mechanistic basis for MRGPRX2-targeted precision therapies. However, most current studies rely on rodent models and short-term pharmacological evaluations. The absence of cross-species validation—particularly in non-human primates and humanized systems—continues to limit the assessment of long-term efficacy, safety, and disease context relevance. These evidence gaps present significant obstacles to the clinical translation of MRGPRX2-targeted interventions.

## 2 Methodology

This narrative mini-review incorporated systematic elements to ensure comprehensive coverage and critical integration. Literature searches were conducted in PubMed and Web of Science and supplemented by targeted queries in Google Scholar, covering publications from 2017 to 2025. Search terms included combinations of “mast cells,” “functional heterogeneity,” “MRGPRX2,” “neuroimmune crosstalk,” “chronic spontaneous urticaria,” “atopic dermatitis,” “allergic contact dermatitis,” and “precision medicine.” Boolean operators (e.g., AND, OR) were applied to optimize search breadth and relevance. Only peer-reviewed original articles and reviews addressing mechanistic insights or translational relevance were included. Non-peer-reviewed, non-English, or thematically unrelated publications were excluded. The primary objective was to synthesize emerging evidence on mast cell heterogeneity and MRGPRX2-associated pathways and to position these findings within evolving frameworks for precision-targeted therapy.

## 3 Functional heterogeneity of MCs in cutaneous inflammation

MCs are tissue-resident immune cells of bone marrow origin (9). They are traditionally categorized into two primary subtypes based on granule protease expression: MC_TC_ (tryptase^+^ chymase^+^), predominantly found in cutaneous connective tissue, and MC_T_ (tryptase^+^ chymase^-^), primarily localized to mucosal compartments (10, 11).

However, this dichotomous classification does not adequately capture the phenotypic plasticity and functional heterogeneity of MCs under inflammatory conditions. Recent single-cell transcriptomic analyses have delineated six high-confidence MC subsets (MC1–MC6) (12). Among them, MC2 exhibits a pro-inflammatory profile, characterized by high expression of IL-13 and prostaglandin-endoperoxide synthase 2, whereas MC4 is enriched for genes such as vascular endothelial growth factor A (VEGFA), colony-stimulating factor 1 (CSF1), interferon gamma receptor 1 (IFNGR1), and interleukin-18 receptor 1 (IL18R1), suggesting roles in angiogenesis and immune regulation (12).

Functional diversity becomes more pronounced in disease-specific contexts. Transcriptomic analysis has revealed an accumulation of cathepsin B-positive MCs (CTSB^+^ MCs) in psoriatic lesions, whose density correlates positively with Psoriasis Area and Severity Index (PASI) scores (13). In autoimmune blistering diseases such as bullous pemphigus, VEGFA^+^ MCs, which express VEGFA, CXCL8, and C5AR1, have been implicated in vascular activation and leukocyte recruitment. In contrast, TNF^+^ MCs, enriched in stress-responsive genes such as TNF, HSPA1B, and FOS, are thought to mediate tissue damage and amplify inflammatory cascades (14). Collectively, these findings underscore the subtype specificity, spatial heterogeneity, and microenvironment-driven phenotypic remodeling of MCs across cutaneous inflammatory disorders. Nonetheless, current nomenclature systems remain inconsistent, and spatial transcriptomic approaches lack methodological standardization. Cross-validation using multicenter datasets and harmonized analytical pipelines is urgently needed. Developing high-throughput, spatiotemporally resolved platforms remains a critical challenge in the study of chronic inflammatory skin diseases.

The functional specialization of MC subpopulations is closely associated with their tissue-specific localization. In human skin, the MCs express high levels of MRGPRX2, which is activated by substance P (SP) to mediate non-IgE-dependent neurogenic pruritus (15). Under chronic inflammatory conditions, MCs co-localize with sensory nerve terminals to form tightly integrated neuroimmune units that contribute to feedback regulation (15). In a contact hypersensitivity (CHS) model in C57BL/6 mice, MCs have been shown to enhance CD8^+^ T cell activation via MHC class I-restricted antigen presentation, while concurrently limiting effector responses through PD-L1-mediated suppression (16). This dual role—promoting cytotoxic responses while restraining excessive inflammation—illustrates the complex pro-inflammatory and immunomodulatory capacities of MCs in cutaneous immunity.

Beyond phenotypic diversity, MC function is modulated by tissue-specific microenvironmental signals and epigenetic mechanisms. Proteomic analyses have demonstrated that MCs from various tissues (e.g., skin and adipose tissue) share core lineage markers, including KIT and CPA3. In contrast, local cues induce differential protein expression, including fatty acid–binding protein 4 (FABP4) and MRGPRX2, which are selectively expressed in adipose tissue and skin, respectively (17). Interleukin-33 (IL-33) amplifies MC activation through FcϵRI and MRGPRX2 signaling cascades—primarily p38/NF-κB and ERK/PI3K—thereby enhancing mediator release, prolonging kinase activation, and dynamically tuning activation thresholds according to microenvironmental conditions (18). Epigenetic regulation further contributes; DNMT3A-mediated DNA methylation suppresses inflammatory activity (19), while histone deacetylase (HDAC) inhibitors such as valproic acid attenuate FcϵRI expression (20) and reduce IL-6 production (21). Additionally, deletion of the solute carrier Slc37a2 disrupts intracellular glucose-6-phosphate (G6P) homeostasis, impairs mTORC1-dependent granule protein synthesis, and limits regranulation capacity (22).

Collectively, these findings support a “nature–nurture” model in which MC phenotypes are determined by intrinsic developmental programs shaped by extrinsic neural, immune, metabolic, and epigenetic inputs (23). However, most available data are derived from animal or *in vitro* models. The lack of longitudinal lineage tracing and human-specific functional validation remains a significant barrier to the clinical translation of these mechanistic insights into MC-targeted precision therapies.

## 4 MRGPRX2-mediated non-IgE activation and its central role in cutaneous inflammation

**Figure 1:**
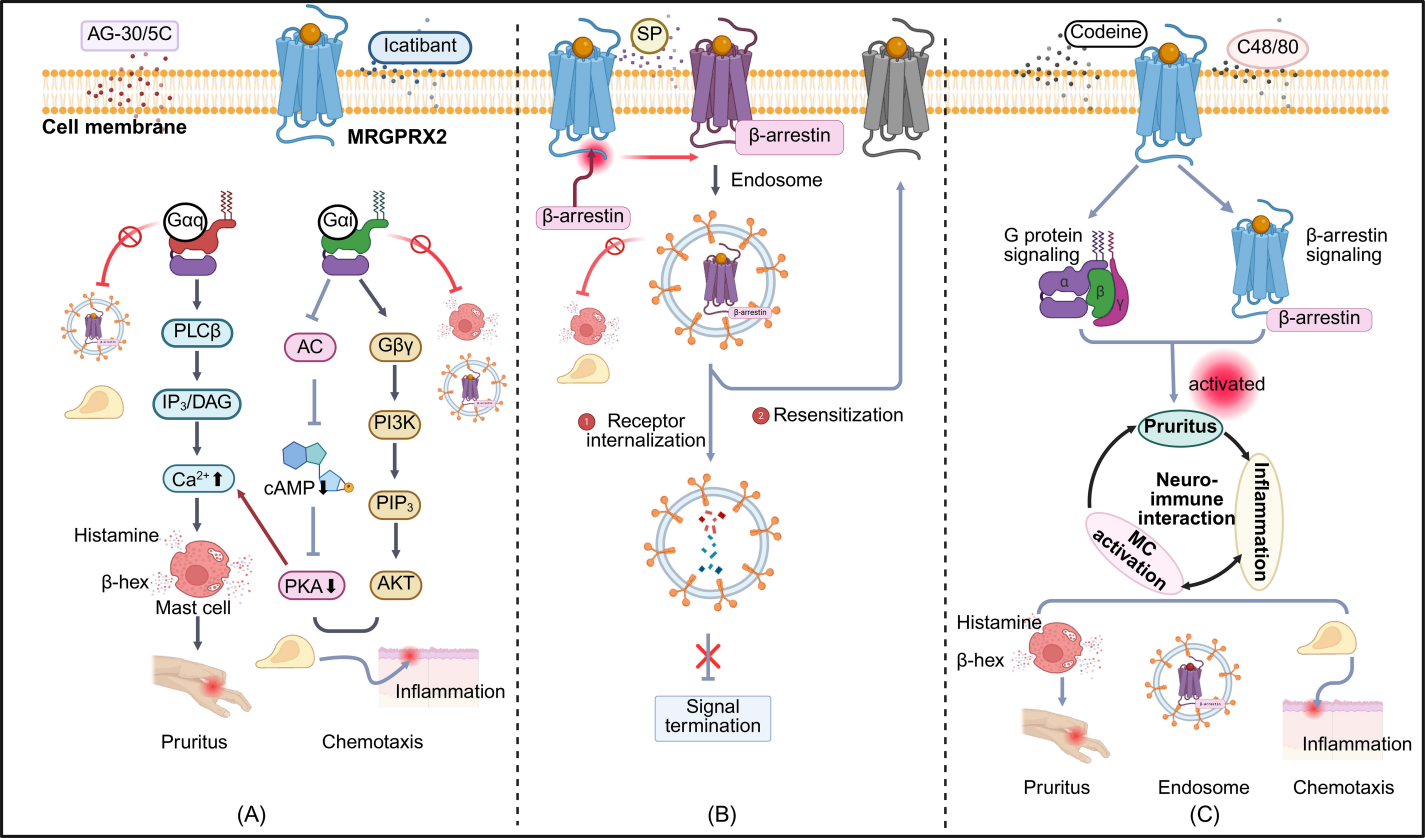
Biased signaling pathways of MRGPRX2 in mast cell responses. Three distinct modes of MRGPRX2-biased agonism are depicted: **(A)** G protein-biased activation: Ligands such as AG-30/5C and Icatibant preferentially activate G protein-mediated signaling. Gaq-PLCB-IP3/DAG activation induces intracellular Ca2+ influx and mast cell degranulation, leading to the release of histamine, ß-hexosaminidase, and other proinflammatory mediators that trigger neurogenic inflammation and pruritus. The Gai-PI3K-AKT pathway promotes chemotaxis by reducing intracellular cAMP levels through inhibition of adenylate cyclase. Suppression of PKA further enhances Ca2+ influx and amplifies Gaq-dependent degranulation. **(B)** β-arrestin-biased activation: Agonists such as SP preferentially recruit ß-arrestins, facilitating receptor conformational changes and internalization. Internalized receptors are either degraded via lysosomes or recycled to the plasma membrane for reactivation. **(C)** Balanced agonism: Codeine and C48/80 simultaneously activate G protein-and β-arrestin–mediated pathways, resulting in degranulation, chemotaxis, and receptor internalization. These responses are implicated in chronic pruritus and cutaneous inflammation. PLCB, Phospholipase C beta ; IP3, Inositol 1,4,5-trisphosphate; DAG, Diacylglycerol; AC, Adenylate Cyclase; cAMP, Cyclic Adenosine Monophosphate; PKA, Protein Kinase A; Gßy, G beta-gamma subunit; PI3K, Phosphoinositide 3-Kinase; PIP3, Phosphatidylinositol (3,4,5)-trisphosphate; AKT, Protein Kinase B; SP, Substance P; C48/80, Compound 48/80. Symbols: ↑ increase; ↓ decrease; → activation; ¬ inhibition. The figure was created in https://BioRender.com.

MRGPRX2, a central mediator of non-IgE-dependent MC activation, contributes to chronic cutaneous inflammation through its broad recognition of cationic ligands, including SP, the host-defense peptide LL-37, and pharmacological agents such as morphine (24). Cryo-EM analysis revealed shallow, solvent-accessible pockets that accommodate structurally and electrostatically diverse ligands. Key acidic residues (Glu164, Asp184, Asp254) mediate electrostatic interactions, while hydrophobic contacts enhance stability. This conformational flexibility underlies its broad ligand specificity (25). Upon activation, MRGPRX2 triggers G protein signaling and recruits β-arrestin, leading to receptor internalization. This serves as a regulatory mechanism to modulate receptor responsiveness (26). Notably, ligand-specific biased signaling has been observed, whereby distinct ligands preferentially engage divergent downstream pathways. For example, Icatibant and AG-30/5C function as G protein–biased agonists that induce MC degranulation via MRGPRX2, without engaging β-arrestin recruitment or triggering receptor internalization (27). In contrast, compound 48/80 and codeine function as balanced agonists, concurrently triggering both G protein–and β-arrestin–mediated pathways and promoting receptor desensitization (**Figure 1**) (28). This phenomenon of conformational–functional coupling, in which ligand-induced conformational states determine specific signaling outputs, may underlie the divergent biological effects of individual ligands (29). However, the relevance of these mechanisms within inflamed tissues remains to be clarified through integrated structural and functional profiling.

To elucidate the pathophysiological relevance of MRGPRX2 signaling, animal models have been employed to characterize its functional roles. Mas-related G-protein-coupled receptor member B2 (MrgprB2), the murine ortholog of human MRGPRX2, mediates non-histaminergic itch in models of allergic contact dermatitis (ACD). In MrgprB2-deficient mice, contact allergen-induced scratching is markedly attenuated, implicating this receptor in hapten-induced pruritus (30, 31). Importantly, although MrgprB2 is the functional murine ortholog of human MRGPRX2, the two receptors display markedly different sensitivities to SP, with a 360-fold difference in EC_50_ values (mouse: 54 μM; human: 152 nM) (32). These findings underscore the importance of cautious interpretation of rodent data and highlight the need for validation in humanized models to ensure translational relevance in drug screening and therapeutic development targeting MRGPRX2.

Beyond species-specific divergence, MRGPRX2 signaling is subject to dynamic modulation by the inflammatory microenvironment. In psoriatic lesions, MCs exhibit increased spatial proximity to CGRP^+^ nerve fibers, accompanied by upregulated expression of TACR1 (neurokinin-1 receptor), indicating enhanced sensitivity to neuropeptide-mediated signaling (13). Similarly, in AD, engagement of MRGPRX2 by SP induces tryptase release and triggers PAR-2–dependent, histamine-independent neurogenic inflammation (33, 34). This excitatory pathway is negatively regulated by both cytokine-mediated and neuromodulatory mechanisms. Chronic exposure to IL-33 suppresses MRGPRX2 transcription and surface expression in MCs, suggesting a cytokine-driven feedback loop that attenuates receptor responsiveness under persistent inflammatory conditions (35). Concurrently, the endocannabinoid system imposes an additional inhibitory influence: Cannabinoid receptor type 1 (CB1) and CB2 receptors, expressed on MCs and peripheral nerve terminals, dampen neuronal excitability, neuropeptide release, and mast cell degranulation, thereby constituting an intrinsic homeostatic mechanism that restrains sustained MRGPRX2-dependent activation (34). This equilibrium between excitatory and inhibitory neuroimmune signals is further perturbed by microbial stimuli. In a murine model of AD, *Staphylococcus aureus* δ-toxin induces IL-4–dependent, MRGPRX2-mediated β-hexosaminidase release from MCs in an IgE-independent manner—an effect abrogated by the selective MRGPRX2 antagonist QWF (36). These observations implicate microbial dysbiosis as a driver of heightened MRGPRX2-dependent activation, linking epithelial barrier disruption to neuroimmune dysregulation and non-histaminergic itch.Notably, although disease-specific triggers—such as neuropeptide sensitization in PsO, microbial dysbiosis in AD, and hapten-induced pruritus in ACD—differ in etiology, these mechanisms converge on a shared MRGPRX2–neuroimmune axis. This common pathway underscores the pivotal role of MRGPRX2 in chronic cutaneous inflammation and supports its therapeutic targeting in histamine-refractory dermatoses.

## 5 Recent advances in precision-targeted therapies for MCs

### 5.1 Small molecule targeting strategies

Small molecules have emerged as a promising approach for modulating IgE-independent MC activation due to their favorable tissue distribution, oral bioavailability, and structural tunability (37). Several MRGPRX2-targeted agents have demonstrated preclinical activity and translational potential. EP262, the first selective oral MRGPRX2 antagonist to enter Phase II clinical trials, inhibited MC degranulation in preclinical models in a dose-dependent manner. Phase I trials reported a favorable safety profile, with no dose-limiting toxicity and only transient headache and mild elevations in alanine aminotransferase (ALT). EP262 is currently being evaluated in ongoing Phase II trials in patients with CSU (CALM-CSU, NCT06077773), chronic inducible urticaria (NCT06050928) and AD (NCT06144424) (38). GE1111, a small molecule identified through structure-based optimization, selectively inhibited MRGPRX2-mediated degranulation and attenuated the release of monocyte chemoattractant protein-1 (MCP-1) and prostaglandin D_2_ (PGD_2_) (39). C9, the first reported MRGPRX2-selective inverse agonist, effectively blocked both basal and ligand-induced G protein/β-arrestin-dependent signaling and inhibited degranulation induced by SP, PAMP-12, and rocuronium in primary human MCs and LAD2 cells (26). Notably, C9 showed minimal cross-reactivity with FcϵRI or murine MrgprB2, confirming high target specificity. PSB-172656 has been shown to selectively inhibit MRGPRX2-mediated MC degranulation without affecting IgE–FcϵRI–dependent responses (40). Compound B (GSK) inhibited SP-induced tryptase and histamine release in human skin MCs and demonstrated anti-pruritic efficacy in a humanized MRGPRX2 mouse model (41). Clarithromycin, a clinically approved macrolide, was found to modulate the FcϵRI/MRGPRX2–Ca²^+^/MAPK axis via activation of the inhibitory receptor CD300f, supporting its potential for repurposing in MC-driven disorders (15, 42).

In parallel, several histamine H_4_ receptor (H_4_R) antagonists have shown dual anti-pruritic and anti-inflammatory effects, although clinical development remains limited (43). JNJ-39758979 demonstrated a trend toward itch reduction in a Phase IIa trial for AD, but development was discontinued due to off-target neutropenia (44). ZPL-3893787 achieved a 50% reduction in Eczema Area and Severity Index (EASI-50) but did not meet statistical significance for itch improvement (45). Izuforant (LEO 152020), a next-generation H_4_R antagonist, exhibited favorable pharmacokinetics and dose-dependent eosinophil suppression in Phase I trials (46). However, in a randomized, double-blind Phase IIa study for cholinergic urticaria (NCT04853992), it failed to meet the primary endpoint, with significance observed only in the Physician Global Assessment of Severity (PGA-S) (47).

Despite promising early-phase data, the pharmacodynamic mechanisms of these agents remain incompletely characterized, particularly regarding the balance between direct receptor antagonism and downstream immunomodulation. Moreover, current approaches do not leverage the functional heterogeneity of MCs to achieve subpopulation-specific targeting—for example, the previously mentioned histone B^+^ MCs in PsO or VEGFA^+^ MCs in aspergillosis. Most agents also lack long-term safety data. The observed dissociation between clinical endpoints—such as improvement in EASI without corresponding itch relief—highlights the need for more mechanistically informative biomarkers. These limitations collectively hinder the clinical translation of MC-targeted precision therapies.

### 5.2 Antibody-based therapies and nanodelivery platforms

Antibody-based therapies targeting sialic acid-binding immunoglobulin-like lectin 8 (Siglec-8) and Siglec-6 represent both a clinically validated approach and a promising emerging strategy for MC-directed interventions. Siglec-8 is an inhibitory receptor predominantly expressed on the surface of human MCs and eosinophils. Its selective activation induces eosinophil apoptosis and suppresses MC degranulation, thereby exerting anti-inflammatory and immunomodulatory effects (48, 49). Lirentelimab (AK002), a humanized monoclonal antibody against Siglec-8, has demonstrated clinical efficacy in a randomized, placebo-controlled Phase II trial for eosinophilic gastritis, as well as in an open-label proof-of-concept study involving patients with antihistamine-refractory chronic spontaneous and inducible urticaria (50, 51). The agent exhibited a generally favorable safety profile, with mild to moderate infusion-related reactions reported in approximately 60% of participants, and no serious immunotoxicity was observed (50). However, the frequency of infusion reactions underscores the need for improved immunogenicity and delivery optimization.

In contrast, Siglec-6 has emerged as a promising target for MC modulation with a distinct molecular mechanism. Unlike Siglec-8, Siglec-6 forms broader signaling complexes with both the α and β subunits of the high-affinity IgE receptor (FcϵRI) and directly interacts with the mature form of the stem cell factor receptor (KIT), thereby suppressing stem cell factor (SCF)–induced MC activation (52). This expanded protein interaction network enables Siglec-6 to regulate activation pathways beyond immune receptor tyrosine-based activation motif (ITAM)–dependent signaling, particularly the KIT/SCF axis (52). These mechanistic features suggest that Siglec-6 may offer broader regulatory capacity and enhanced target specificity, providing a rationale for the development of more selective therapeutic strategies.

To overcome the limitations of antibody delivery in peripheral tissues such as the skin—including poor tissue penetration, off-target effects, and systemic toxicity—nanodelivery platforms are increasingly being investigated to enhance the precision and efficacy of MC-targeted therapies. A recent study published in Nature Nanotechnology reported the development of a bispecific Siglec-6/FcϵRIα antibody–coated nanoparticle system (53). This engineered platform significantly reduced CD63 surface expression and degranulation in a humanized MC model, providing functional validation of Siglec-6 as a potent inhibitory target. These findings also underscore the potential of nanocarrier-based systems to enable tissue-specific targeting and functional modulation of MCs.

## 6 Prospects for precision medicine research on MCs

In inflammatory skin diseases, MCs demonstrate substantial subpopulation heterogeneity, diverse activation mechanisms, and complex therapeutic response profiles. Beyond their classical role as IgE-dependent effector cells, MCs are increasingly recognized as context-dependent immunomodulators that coordinate neuroimmune interactions and regulate sensory pathways associated with pruritus, pain, and inflammation. Nonetheless, several key barriers hinder clinical translation. These include the lack of dynamic tools for identifying MC subsets, incomplete mapping of regulatory signaling networks, limited translatability of current animal models, and inconsistent standards for biomarker detection across studies. Future efforts should aim to systematically address these challenges to establish scalable and clinically actionable MC-targeted precision strategies.

### 6.1 From static classification to dynamic trajectory analysis

Current investigations of MCs predominantly rely on tissue samples collected at single time points, limiting the ability to capture dynamic transitions in MC phenotypes during the initiation, progression, and resolution of inflammation. For instance, a neurogenic inflammatory microenvironment was simulated by continuously supplementing cultures with SP, resulting in the gradual enrichment of MRGPRX2^+^ MCs *in vitro (* 10). This observation indicates that microenvironmental cues dynamically regulate MC phenotypes. In pathological settings, single-cell transcriptomics, RNA velocity, and spatial transcriptomics have been applied to delineate trajectories from quiescent to VEGFA^+^ activated MC subpopulations. Activated MCs were predominantly localized at the tumor–normal interface and colocalized with IL1B^+^ macrophages, indicating that local inflammatory cues can drive *in situ* phenotypic reprogramming. These observations illustrate that MC phenotypes are not only spatially heterogeneous but also subject to dynamic modulation within disease-specific microenvironments, highlighting the need for spatiotemporally resolved tracking strategies in future research (54). Future studies should utilize technologies with high spatial and temporal resolution to comprehensively map MC subpopulation dynamics. Promising strategies include spatial transcriptomic platforms such as multiplexed error-robust fluorescence *in situ* hybridization (MERFISH) and expansion microscopy (ExM), along with *in vivo* optogenetic tools, to construct a spatiotemporal-functional framework for delineating MC state transitions. Furthermore, integrating longitudinal clinical cohorts with multi-omics datasets—including transcriptomic, epigenomic, and proteomic layers—may facilitate the identification of key environmental regulators (e.g., Slc37a2, TET2) involved in MC phenotypic remodeling (22, 55). Such approaches are essential for elucidating how genetic predisposition and environmental exposures interact to shape MC functional plasticity in chronic inflammatory conditions.

### 6.2 Mechanistic framework for signaling pathway intervention

MRGPRX2, a multifunctional G protein–coupled receptor (GPCR), activates both G protein–dependent signaling and β-arrestin–mediated endocytosis (29). This bifurcated signaling profile provides opportunities for selective therapeutic modulation while complicating pathway-specific regulation (26). Future research combining cryo-electron microscopy and molecular dynamics simulations is expected to elucidate the structural determinants that link ligand binding to downstream signaling outcomes, thereby facilitating the rational design of biased ligands. A naturally occurring missense variant of MRGPRX2 (V282M) has been shown to exhibit a loss-of-function phenotype for G protein–mediated degranulation while retaining constitutive β-arrestin activity, which is significantly inhibited by the inverse agonist C9 (26). However, their impact on antagonist binding and pharmacodynamic responses remains undefined. A multicenter genetic cohort study is warranted to establish correlations between mutational profiles and therapeutic responses. These efforts will be instrumental in developing a mutation–function atlas to guide individualized treatment strategies.

### 6.3 Establishing a closed-loop framework for high-fidelity experimental and functional evaluation

Establishing a high-fidelity humanized experimental model is essential for accurately delineating the role of the human MRGPRX2 pathway in MC-mediated pathological responses. Current platforms—including MRGPRX2 knock-in (KI) mice generated via genetic modification (56), bone marrow-derived mast cells (BMMCs) ectopically expressing the human receptor (9), and humanized models derived from hematopoietic stem cells (57) —only partially replicate native human MRGPRX2 functionality. Each exhibits critical limitations: KI mice differ in ligand sensitivity and signal transduction fidelity; BMMCs lack appropriate tissue distribution and maturation profiles; and hematopoietic stem cell–derived models often show restricted receptor expression in specific tissues, limiting mechanistic extrapolation. Recent insights further underscore the complexity of modeling MRGPRX2-mediated signaling in human dermatoses such as AD and ACD. In these contexts, receptor activity is influenced not only by neuropeptides but also by microbial metabolites and chronic cytokine exposure—factors that are challenging to recapitulate in conventional rodent systems (31, 58). These multifactorial inputs modulate MRGPRX2 expression and alter downstream inflammatory mediator profiles, thereby limiting the extrapolative validity of existing preclinical models. To overcome these constraints, future experimental models should more faithfully recapitulate the phenotypic and functional characteristics of human MCs, thereby enhancing their translational applicability to clinical research. One strategy involves CRISPR/Cas9-mediated generation of humanized MRGPRX2 knock-in mouse models, which preserve native tissue architecture while enabling *in vivo* assessment of human-specific signaling (59). Alternatively, MCs derived from induced pluripotent stem cells (iPSCs) and co-cultured with three-dimensional skin or intestinal organoids (60) can simulate immune-tissue interactions, allowing for the dynamic evaluation of MC responses to environmental stimuli. In addition, emerging MC-microbiota co-culture models have provided insights into the microbiome-MC axis in chronic inflammation and immune homeostasis, offering platforms for the development of complex comorbidity models.

Despite advances in model fidelity, the evaluation of MC-targeted interventions remains limited by the lack of standardized functional metrics and validated detection methods. MRGPRX2 expression and serum levels of its ligands, such as SP, have been proposed as potential biomarkers of MC activation (38). Emerging evidence indicates that serum MRGPRX2 levels positively correlate with disease activity (UAS7) in treatment-naïve patients with CSU, and elevated SP levels have also been observed in CSU cohorts (61, 62). However, substantial variability in sample types (e.g., biopsy vs. microdialysate) and assay platforms (e.g., ELISA vs. Simoa) limits cross-study comparability. Functional MRGPRX2 variants, including gain-of-function mutations such as N16H, N62S, and S313R—identified in patients with hypersensitivity to quinolones and vancomycin—have been shown to enhance MC activation in a drug-specific manner (63). These findings highlight the role of MRGPRX2 genetic variability in modulating pharmacodynamic responses to specific drugs and underscore the need for mechanism-based diagnostic strategies to predict and manage hypersensitivity reactions.

A unified functional assessment system is therefore warranted, incorporating metrics such as degranulation kinetics, cytokine profiles, signaling activation efficiency, and clinical endpoints (e.g., pruritus severity and quality-of-life indices) to enhance the translational coherence between basic and clinical research. Strategically, these efforts should be aligned with the development of multicenter prospective cohorts and dermatology biobanks, enabling longitudinal monitoring of therapeutic efficacy, safety, and reversibility. Ultimately, translational pipelines must evolve beyond single-model, single-marker paradigms toward multi-scale, scenario-specific validation systems that bridge mechanistic insight with clinical outcomes and support the implementation of personalized MC-targeted therapies.
